# Negative Regulation of *Schistosoma japonicum* Egg-Induced Liver Fibrosis by Natural Killer Cells

**DOI:** 10.1371/journal.pntd.0001456

**Published:** 2012-01-03

**Authors:** Xin Hou, Fazhi Yu, Suqin Man, Dake Huang, Yuxia Zhang, Miao Liu, Cuiping Ren, Jijia Shen

**Affiliations:** 1 Department of Microbiology and Parasitology, Anhui Provincial Laboratory of Microbiology and Parasitology, Anhui Medical University, Hefei, Anhui, People's Republic of China; 2 College of Basic Medical Sciences, Anhui Medical University, Hefei, Anhui, People's Republic of China; Uniformed Services University, United States of America

## Abstract

The role of natural killer (NK) cells in infection-induced liver fibrosis remains obscure. In this study, we elucidated the effect of NK cells on *Schistosoma japonicum* (*S. japonicum*) egg-induced liver fibrosis. Liver fibrosis was induced by infecting C57BL/6 mice with 18–20 cercariae of *S. japonicum*. Anti-ASGM1 antibody was used to deplete NK cells. Toll-like receptor 3 ligand, polyinosinic-polycytidylic acid (poly I∶C) was used to enhance the activation of NK cells. Results showed that NK cells were accumulated and activated after *S. japonicum* infection, as evidenced by the elevation of CD69 expression and IFN-γ production. Depletion of NK cells markedly enhanced *S. japonicum* egg-induced liver fibrosis. Administration of poly I∶C further activated NK cells to produce IFN-γ and attenuated *S. japonicum* egg-induced liver fibrosis. The observed protective effect of poly I∶C on liver fibrosis was diminished through depletion of NK cells. Disruption of IFN-γ gene enhanced liver fibrosis and partially abolished the suppression of liver fibrosis by poly I∶C. Moreover, expression of retinoic acid early inducible 1 (RAE 1), the NKG2D ligand, was detectable at high levels on activated hepatic stellate cells derived from *S. japonicum*-infected mice, which made them more susceptible to hepatic NK cell killing. In conclusion, our findings suggest that the activated NK cells in the liver after *S. japonicum* infection negatively regulate egg-induced liver fibrosis via producing IFN-γ, and killing activated stellate cells.

## Author Summary

Schistosomiasis continues to be a major public health problem in the developing world. Parasite egg-induced liver fibrosis is the principal cause of morbidity and mortality in human infected with schistosoma. Thus, elucidating the mechanisms that restrict tissue fibrosis may lead to more effective strategies for immunological intervention in this and a variety of chronic diseases. NK cells have been demonstrated to play an important role in suppressing carbon tetrachloride (CCl_4_)-induced liver fibrosis. However, little is known about the role of NK cells in an infection-based model of fibrosis. In the current study, we determined, for the first time, the role of NK cells in *S. japonicum* egg-induced liver fibrosis. Our findings suggest that the activated NK cells in the liver after *S. japonicum* infection negatively regulate egg-induced liver fibrosis via producing IFN-γ, and killing activated stellate cells. These results further our understanding of the innate immune cells that regulate the development of *S. japonicum*-induced fibrosis and aid in the development of potential strategies to enhance immunity against this and other chronic inflammatory diseases of the liver where fibrosis is a common feature.

## Introduction

The development of fibrous tissue is a common scarring response to chronic and debilitating illnesses, including several autoimmune, allergic, and infectious diseases [1]. In schistosomiasis, egg-induced hepatic fibrosis can lead to portal hypertension, which causes much of the morbidity and mortality associated with this disease. Murine models of schistosome infection indicate that most pathology is attributable to type 2 T-helper cell (Th2)-mediated granulomatous response against schistosome eggs and egg antigens [2]. These Th2 cytokines, in particular IL-13 and its receptor (IL-13Rα), play a pivotal role in the development of granuloma-associated fibrosis [3], [4]. In addition, anti-IL-4 or anti-IL-5 treatment markedly decreases schistosomiasis-induced fibrosis [5], [6]. IFN-γ has major roles in downregulating Th2 cell expansion [7] and selectively promoting Th1 cell differentiation [8]. Moreover, IFN-γ has been shown to have potent antifibrogenic effect [9]–[11], and its antifibrogenic effect is believed to be mediated via inhibiting hepatic stellate cell (HSC) activation and TGF-β signaling [12], [13]. In *Schistosoma mansoni (S. mansoni)*-infected mice, IFN-γ treatment leads to significant reduction of hepatic fibrosis [14], [15].

Natural killer (NK) cells represent a major source of IFN-γ [16]–[18]. The antifibrogenic roles of these cells have been studied in two artificial models of liver fibrosis induced by a 3,5-diethoxycarbonyl-1,4-dihydrocollidine diet (DDC) and carbon tetrachloride (CCl_4_). The authors showed that hepatic NK cells killed activated HSCs via retinoic acid early inducible 1 (RAE1)/NKG2D dependent and tumor necrosis factor-related apoptosis-inducing ligand (TRAIL)-dependent mechanisms, thereby inhibited liver fibrosis [19]. Little is known about the roles of NK cells in an infection-based model of fibrosis. NK cells have been demonstrated within schistosome egg granulomas [20]. However, the roles of NK cells in the development and progression of schistosome egg-induced liver fibrosis remain unknown. Here, in *S. japonicum*-infection model, we found that hepatic NK cells markedly ameliorated egg-induced liver fibrosis. NK cells played their critical roles partly via an IFN-γ-dependent mechanism. We also presented evidence that NK cells might kill activated HSCs to attenuate fibrosis via NKG2D-RAE1 recognition.

## Materials and Methods

### Ethics Statement

All mice were maintained in a specific pathogen-free microenvironment, and received care in compliance with the guidelines outlined in the *Guide for the Care and Use of Laboratory Animals*. All work was conducted with the approval of the Anhui Experimental Animal Training Base.

### Animals, parasites, and infections

Six-week old female C57BL/6 mice were purchased from Experimental Animal Center, Chinese Science Academy (Shanghai, China). Interferon-gamma knockout (IFN-γ−/−) mice were purchased from model animal research central of Nanjing University. For infection, mice were anesthetized and percutaneously exposed to 18–20 cercariae of *S. japonicum* (strain from Jiangxi Province, China) that were obtained from infected *Oncomelania hupensis* snails.

### Depletion of natural killer cells by anti-ASGM1 antibody

To deplete NK cells, mice were injected intravenously with anti-ASGM1 antibody (Ab) (Wako Co., Tokyo, Japan). After 24 hours, depletion of NK cells was confirmed by flow cytometry. To chronically deplete NK cells, mice were treated with anti-ASGM1 Ab every 5 days from week 5 after infection for 3 or 5 weeks.

### Analysis of liver transaminase activities

Serum samples from individual mice were obtained at week 8 and week 10 post-infection. Liver injury was assessed by measuring serum alanine aminotransferase (ALT) activities using commercially available kit (Rong Sheng, Shanghai, China).

### Treatment of mice with polyinosinic∶polycytidylic acid

Poly I∶C (Sigma Chemical Co., St Louis, MO) was dissolved in the pyrogen-free saline. Mice were injected intraperitoneally with poly I∶C (0.5 µg/g) every 3 days since week 5 post-infection. Control infected mice received saline injection.

### Histology and immunohistochemistry

Liver tissues were fixed in 10% buffered formalin and embedded in paraffin. Tissue sections were affixed to sides, deparaffinized, and stained with Masson trichrome for collagen deposition. Immunostaining for α-smooth muscle actin (α-SMA) was performed using a monoclonal α-SMA primary Ab (clone 1A4; Dako, Carpinteria, CA), and a horseradish peroxidase-labeled secondary Ab. Six to ten images per mouse liver were photographed using an inverted microscope (Nikon 80I, Japan) and then digitized and analyzed on Image-Pro Plus software.

### Western blot

Liver tissues were homogenized in RIPA lysis buffer (Solarbio, China) added with 1 mM PMSF. Western blot analyses were performed as described previously [21]. Briefly, proteins were separated by 10% SDS-polyacrylamide gel electrophoresis, transferred onto nitrocellulose membranes, and blotted with primary Abs. After wash, membranes were incubated with horseradish peroxidase-conjugated secondary Abs. Specific binding was visualized by ECL reaction (Pierce).

### Isolation of hepatic stellate cells

HSCs were isolated using two-step collagenase perfusion method as described [22]. The viability of the isolated cells was determined to be 98% using trypan blue staining. The purity of the cells was assessed visually by light microscopy examination of typical lipid droplet appearance, and vitamin A autofluorescence was more than 90%.

### Isolation and culture of liver mononuclear cells

Liver mononuclear cells (MNCs) were isolated essentially as described previously [17]. To culture liver MNCs *in vitro*, cell pellets were resuspended in RPMI 1640 medium containing penicillin, streptomycin, and 10% FBS, and then plated onto 24-well plates at a density of 5×10^6^ cells per well in 1 mL culture medium with or without poly I∶C treatment for 48 hours. The supernatants were collected for IFN-γ measurement by ELISA (R&D system).

### Quantitative PCR

The RNA of HSCs was extracted using RNAprep pure Micro Kit (Tiangen Biotech CO., LTD.). Quantitative PCR was performed using a sequence detector (ABI-Prism 7500; Applied Biosystems) and a SYBR Premix Ex Taq (Takara), according to the manufacturer's instructions. Primers for RAE1α, RAE1β, RAE1γ, RAE1δ, RAE1ε, H60, and Mult1 were used as reported previously [19]. Other primer sequences used are as follows: β-actin, sense, 5′-TGG AAT CCT GTG GCA TCC ATG AAA-3′, antisense, 5′-TAA AAC GCA GCT CAGTAA CAG TCC G-3′; IL-4, sense, 5′-TCA TGG AGC TGC AGA GAC TCT T-3′, antisense, 5′-CAT TCA TGG TGC AGC TTA TCG A-3′; IL-5, sense, 5′-CTC TGT TGA CAA GCA ATG AGA CG-3′, antisense, 5′-TCT TCA GTA TGT CTA GCC CCT G-3′; IL-13, sense, 5′- CCT GGC TCT TGC TTG CCT T-3′, antisense, 5′-GGT CTT GTG TGA TGT TGC TCA-3′. For analysis, all expression levels of target genes were normalized to the housekeeping gene β-actin (ΔCt). Gene expression values were then calculated based on the ΔΔCt method as mentioned previously [17].

### Isolation of NK Cells

Liver NK cells were separated by positive magnetic cell sorting using anti-DX5 mAb according to the manufacturer's protocol (Miltenyi Biotec, Auburn, CA) from *S. japonicum*-infected mice. Approximately 90% of the magnetic cell sorting-purified cells were DX5+.

### Flow Cytometric Analysis

The monoclonal Abs used for flow cytometry in this study included Cy5-anti-CD3e, phycoerythrin (PE)-anti-CD69, fluorescein isothiocyanate (FITC)-anti-NK1.1, PE-anti-IFN-γ, PE Rat immunoglobulin G2a (IgG2a) isotype control (BD PharMingen). For intracellular cytokine staining, liver MNCs were incubated for 4 hours in the presence of ionomycin (1 µg/ml, Sigma), PMA (20 ng/ml, Sigma), and brefeldin A (1 µg/ml, BD Pharmingen). After staining with FITC-anti-NK1.1 mAb and Cy-5-anti-CD3e mAb, cells were fixed, permeabilized, and stained with PE-anti-IFN-γ mAb using a Cytofix/Cytoperm plus kit (BD PharMingen). The stained cells were analyzed using a flow cytometer (FACScalibur; Becton Dickinson, Franklin Lakes, NJ), and data were analyzed with WinMDI2.9 software.

### Cytotoxicity assay

NK cell-mediated cytotoxicity against primary HSCs was measured by ToxiLight BioAssay Kit (Lonza, Rockland, Inc). Briefly, freshly isolated HSCs were plated onto round-bottom, 96-well plates. NK cells were subsequently added as effector cells. The release of adenylate kinase from damaged cells was measured from the culture medium by using the ToxiLight kit. Specific lysis was calculated according to the formula [(test release−spontaneous release)/(maximum release−spontaneous release)]×100.

### Statistical Analysis

Data were analysed using SPSS (v11.0) and GraphPad Prism (v5). To compare values obtained from multiple groups, 1-factor analysis of variance (ANOVA) was used, followed by Tukey's post hoc test. To compare values obtained from two groups, the Student t test was performed. All data were shown as mean ± standard error of the mean (SEM). *P* value≤0.05 was considered to be statistically significant.

## Results

### Activation of hepatic NK cells during *S. japonicum* infection

**Figure 1:**
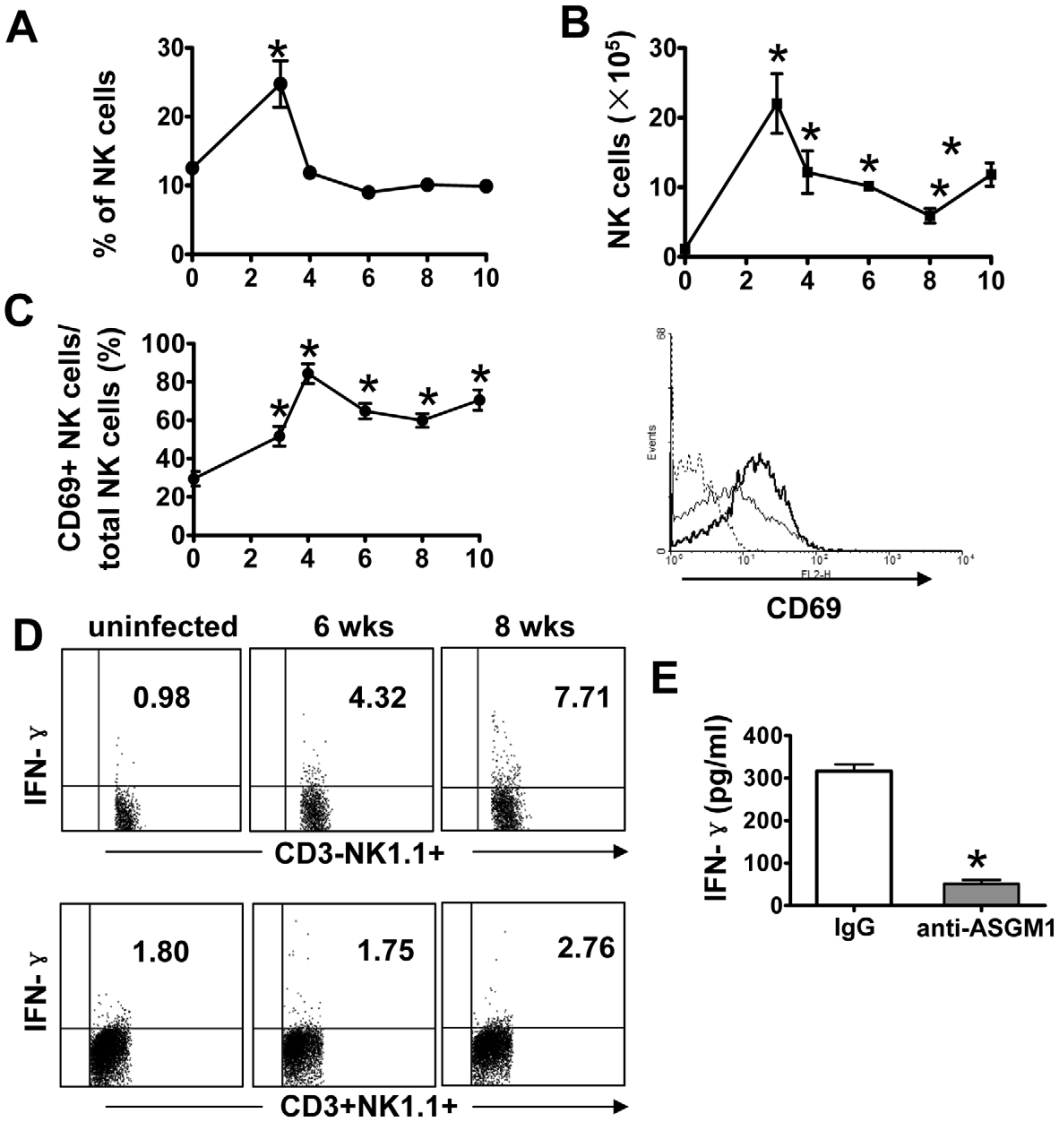
Analysis of hepatic NK cell activation during *S. japonicum* infection. C57BL/6 mice were percutaneously infected with 18–20 cercariae of *S. japonicum*. (A–C) After various time periods post-infection, hepatic MNCs were prepared for flow cytomery analysis. The relative proportion (A) and absolute number (B) of CD3-NK1.1+ cells were shown. (C) Expression of CD69 on hepatic NK cells from uninfected (thin lines) or infected mice (bold lines) during infection (right panel). For the kinetic study, the percentage of CD69+ NK cells among total NK cells was calculated and represented (left panel). Data were shown as mean ± SEM (n = 5 for each group). *, *P*<0.05 versus corresponding 0 week groups. (D) Liver MNCs isolated from uninfected or *S. japonicum*-infected mice, and then were analyzed by flow cytometry for intracellular IFN-γ of NK cells (CD3-NK1.1+) and NKT (CD3+NK1.1+) cells. (E) Hepatic MNCs were isolated from control IgG or anti-ASGM1 pretreated mice at week 6 post-infection. After culture for 48 hours, the supernatants were collected for IFN-γ measurement by ELISA. Data were presented as mean ± SEM; *, *P*<0.05 compared with corresponding control IgG treated group.

To investigate whether NK cells were involved in *S. japonicum* infection-induced liver fibrosis, we first determined the activation of NK cells in the liver post-infection. As shown in Figure 1A, the percentage of NK cells among hepatic MNCs significantly increased at week 3 post-infection, and then diminished to baseline and stabilized between the forth and the tenth week of infection. The absolute number of hepatic NK cells increased dramatically during infection, which peaked at week 3 post-infection (Figure 1B). Furthermore, the percentage of CD69 positive NK cells was significantly increased from week 3 to 10 (Figure 1C).

IFN-γ production of NK cells was also measured. As shown in Figure 1D, at week 6 and week 8 post-infection, the number of NK cells (CD3-NK1.1+) stained positively for IFN-γ was increased. Since NKT cells can also produce IFN-γ, we examined IFN-γ production of these cells. The increase in the number of NKT cells (CD3+NK1.1+) stained positively for IFN-γ after infection was very slight. Moreover, IFN-γ secretion by hepatic MNCs from anti-ASGM1 Ab pretreated mice was strongly reduced compared to that from control IgG pretreated mice (Figure 1 E). These results suggested that IFN-γ was mainly produced by liver NK cells.

### Depletion of NK cells accelerates *S. japonicum* egg-induced fibrosis

**Figure 2:**
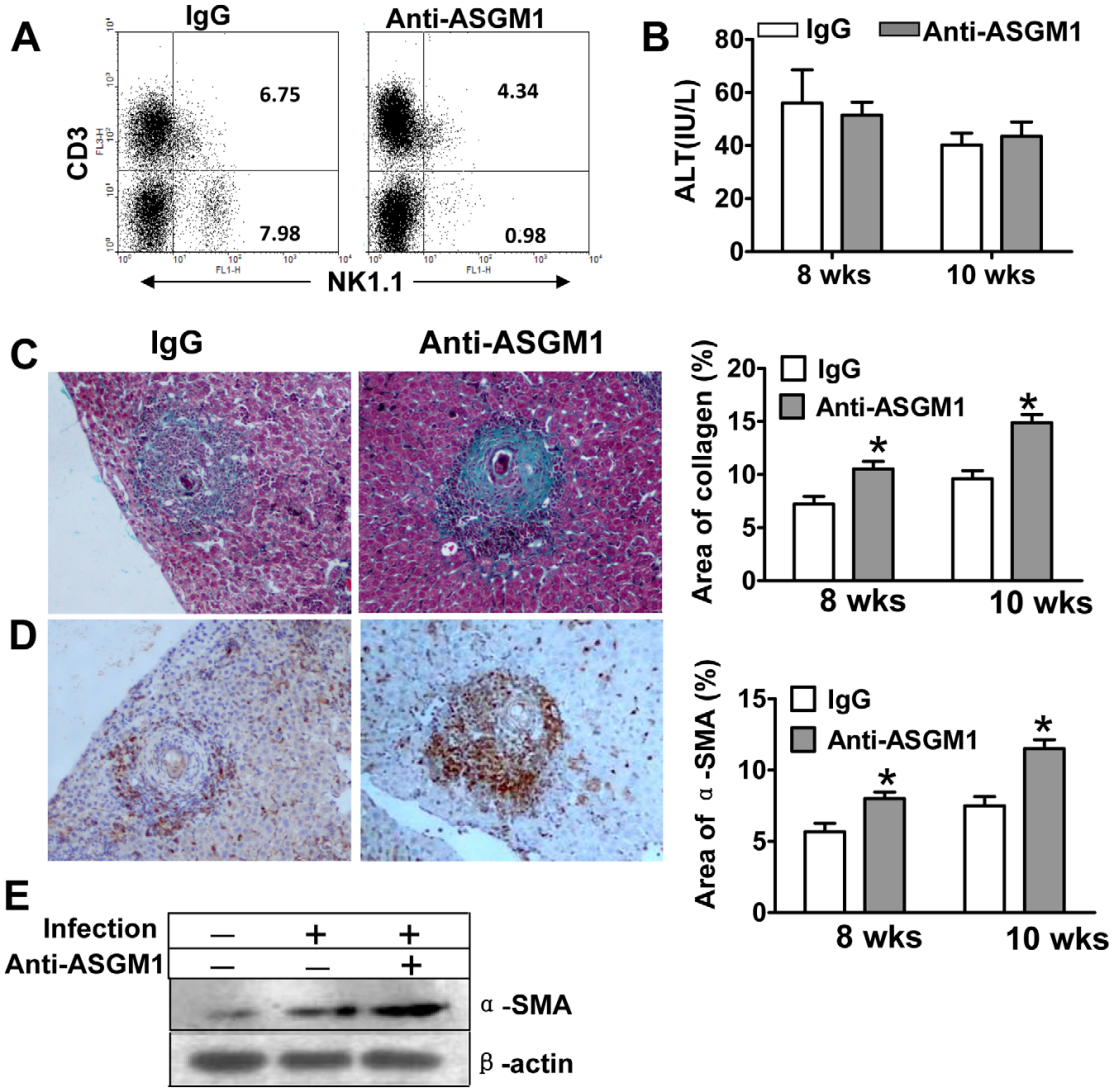
Depletion of NK cells enhances *S. japonicum* egg-induced liver fibrosis. NK cells were chronically depleted by anti-ASGM1 Ab. (A) The efficiency of depletion was verified by flow cytometry. (B) Serum ALT levels were measured at week 8 and week 10 post-infection. Data were shown as means ± SEM from six mice at each time point in each group. Liver tissues were fixed and stained with Masson trichrome (C) or anti-α-SMA antibody (D) (original magnification×100). Collagen deposition and the α-SMA+area were quantified and shown on the right side in (C) and (D), respectively. Data were presented as mean ± SEM from 7 mice per each group. *, *P*<0.05 versus control IgG-treated groups. (E) Liver tissues from 8 week *S. japonicum*-infected mice were subjected to Western blot analyses for α-SMA protein expression.

To investigate the role of NK cells in *S. japonicum* egg-induced liver fibrosis, anti-ASGM1 Ab was used to deplete NK cells for 3 or 5 weeks. The depletion effect was confirmed by flow cytometry (Figure 2A). At week 8 and week 10 post-infection, the liver injury was evaluated by examining serum ALT activities. As shown in Figure 2B, depletion of NK cells did not significantly affect ALT levels. Meanwhile, liver fibrosis was monitored by Masson trichrome staining for collagen deposition (blue staining) and immunohistochemical staining for HSC activation (α-SMA-positive staining). Results showed that depletion of NK cells significantly enhanced *S. japonicum* egg-induced collagen deposition (Figure 2C) and elevation of α-SMA+ cells (Figure 2D). The inhibitory effect of NK cells on HSC activation was also confirmed by Western blot analyses. As shown in Figure 2E, α-SMA protein expression was increased in anti-ASGM1-treated mice compared with IgG-treated mice.

### Poly I∶C injection attenuates *S. japonicum* egg-induced hepatic fibrosis via NK cell-dependent manner

**Figure 3:**
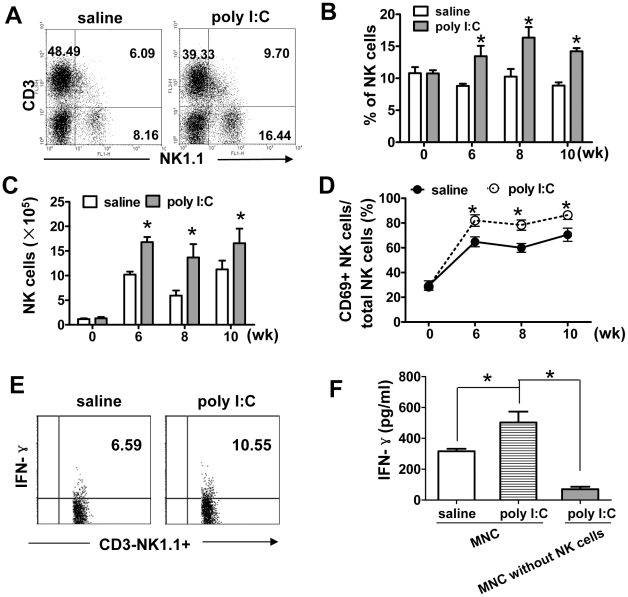
Injection of poly I∶C further activates NK cells in *S. japonicum* infected mice. *S. japonicum*-infected mice were injected intraperitoneally with poly I∶C (0.5 µg/g) since week 5 post-infection. (A–D) At various time points, hepatic MNCs were isolated and subjected for flow cytomery analysis. A representative FACS analysis of NK1.1 and CD3 was shown in (A). The percentage of NK cells among hepatic MNCs and the total number of NK cells are summarized in (B) and (C), respectively. The percentage of CD69+ NK cells among total NK cells was calculated and represented in (D). Data were presented as mean ± SEM (n = 5 for each group). *, *P*<0.05 versus corresponding saline-treated group. (E) Mice were sacrificed at week 6 post-infection, and IFN-γ expression by hepatic NK cells (CD3-NK1.1+) was examined by flow cytometry. (F) Hepatic MNCs from control IgG or anti-ASGM1 pretreated mice (denoted as “MNCs” and “MNCs without NK cells”, respectively) were incubated with poly I∶C (100 µg/ml) or saline for 48 hours. IFN-γ secretion in the supernatant was measured by ELISA. Data were presented as mean ± SEM; *, *P*<0.05.

Poly I∶C could activate NK cells and induce accumulation of NK cells in the liver but did not induce remarkable elevation of ALT in B6 mice [17], [21]. Therefore, we treated infected mice with poly I∶C to study the effect of NK cell activation on liver fibrosis. First, the effect of poly I∶C injection on NK cell activation was examined. As shown in Figure 3(A–C), the percentage and absolute number of NK cells in the liver post-infection were markedly elevated in poly I∶C-treated mice compared with those in saline-treated mice. And poly I∶C injection also increased CD69 expression on NK cells (Figure 3D). Notably, the percentage of IFN-γ positive NK cells was notably increased in poly I∶C-treated group (Figure 3E). Further analyses of IFN-γ concentration in the culture supernatants of hepatic MNCs derived from *S. japonicum* infected-mice showed that stimulation with poly I∶C promoted hepatic MNCs to produce IFN-γ, but not MNCs without NK cells, which further suggested that NK cells were mainly responsible for IFN-γ production (Figure 3F).

**Figure 4:**
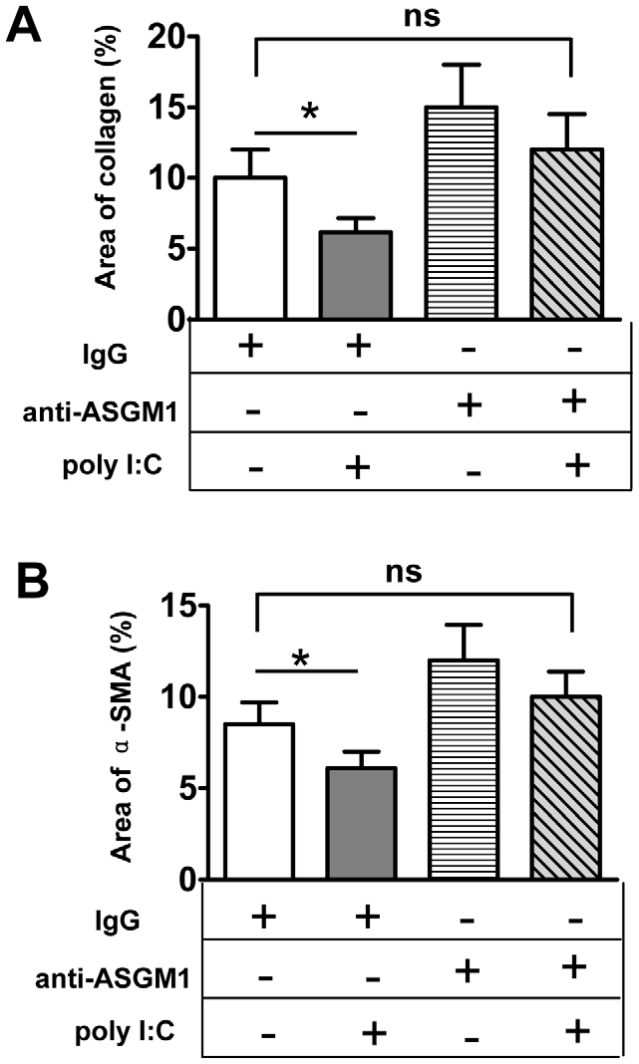
Poly I∶C injection attenuates *S. japonicum* egg-induced hepatic fibrosis via NK cell-dependent manner. *S. japonicum*-infected mice were chronically treated with control IgG or anti-ASGM1 Ab and poly I∶C from week 5 to week 10 post-infection. Collagen deposition and the α-SMA+area were quantified and shown in (A) and (B), respectively. Data were presented as mean ± SEM (n≥6 for each group). *, *P*<0.05; ns, not significant.

Next, the role of poly I∶C on *S. japonicum* egg-induced fibrosis was examined. As shown in Figure 4, poly I∶C treatment significantly reduced *S. japonicum* egg-induced collagen deposition and HSC activation (α-SMA+ cells). However, the inhibitory effect of poly I∶C was abolished in anti-ASGM1-treated mice, which suggested that poly I∶C suppression of liver fibrosis was dependent on NK cells.

### NK cells inhibit *S. japonicum* egg-induced fibrosis via IFN-γ and NKG2D

**Figure 5:**
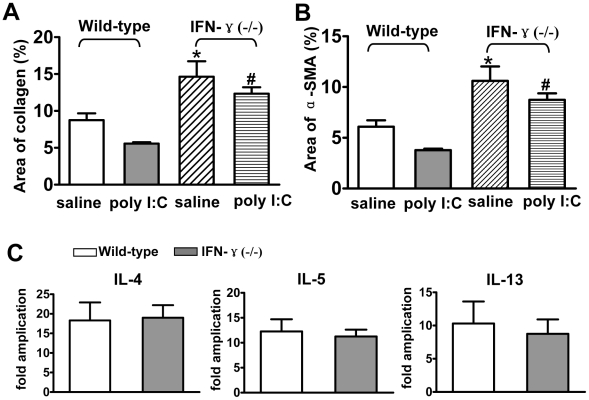
Disruption of the IFN-γ gene accelerates hepatic fibrosis and partially abolish poly I∶C-mediated suppression. (A–C) Wild-type and IFN-γ−/− mice were infected with 18–20 cercariae of *S. japonicum*, and injected with poly I∶C (0.5 µg/g, i.p.) or saline from week 5 to week 10 post-infection. All mice were euthanized at week 10 post-infection. (A–B) Liver tissues were subjected to stain for quantifying collage (A) and α-SMA positive areas (B). Data were shown as mean ± SEM from 7–9 mice per each group. *, *P*<0.05 versus corresponding saline-treated wild-type mice. #, *P*<0.05 versus corresponding poly I∶C-treated wild-type mice. (C) Intrahepatic IL-4, IL-5, and IL-13 mRNA expression levels were measured by quantitative PCR. Results were expressed as fold amplification over normal, uninfected liver following normalization with β-actin. Data were expressed as mean ± SEM (n≥7 for each group).

To examine whether NK cells negatively regulated *S. japonicum* egg-induced liver fibrosis via production of IFN-γ, we compared liver fibrosis between wild-type and IFN-γ−/− mice. As shown in Figure 5A, *S. japonicum* egg-induced liver fibrosis in IFN-γ−/− mice was much more severe than that in wild-type mice (14.65%±4.21 versus 8.76%±1.83, *P* = 0.04). Consonantly, there were more α-SMA positive HSCs in IFN-γ−/− mice than those in wild-type mice (10.60%±2.85 versus 6.10%±1.24, *P* = 0.02) (Figure 5B). These findings suggested that IFN-γ played negative roles in egg-induced liver fibrosis. Moreover, IFN-γ was also involved in poly I∶C-mediated suppression of liver fibrosis. Results showed that the suppression of collagen deposition and HSC activation by poly I∶C in IFN-γ−/− mice was reduced compared with that in wild-type mice (Figure 5A, B). Furthermore, we examined the Th2 cytokine expression in the liver of IFN-γ−/− mice. As shown in Figure 5C, IL-4, IL-5 and IL-13 mRNA expression was not different in IFN-γ−/− mice compared with wild mice.

**Figure 6:**
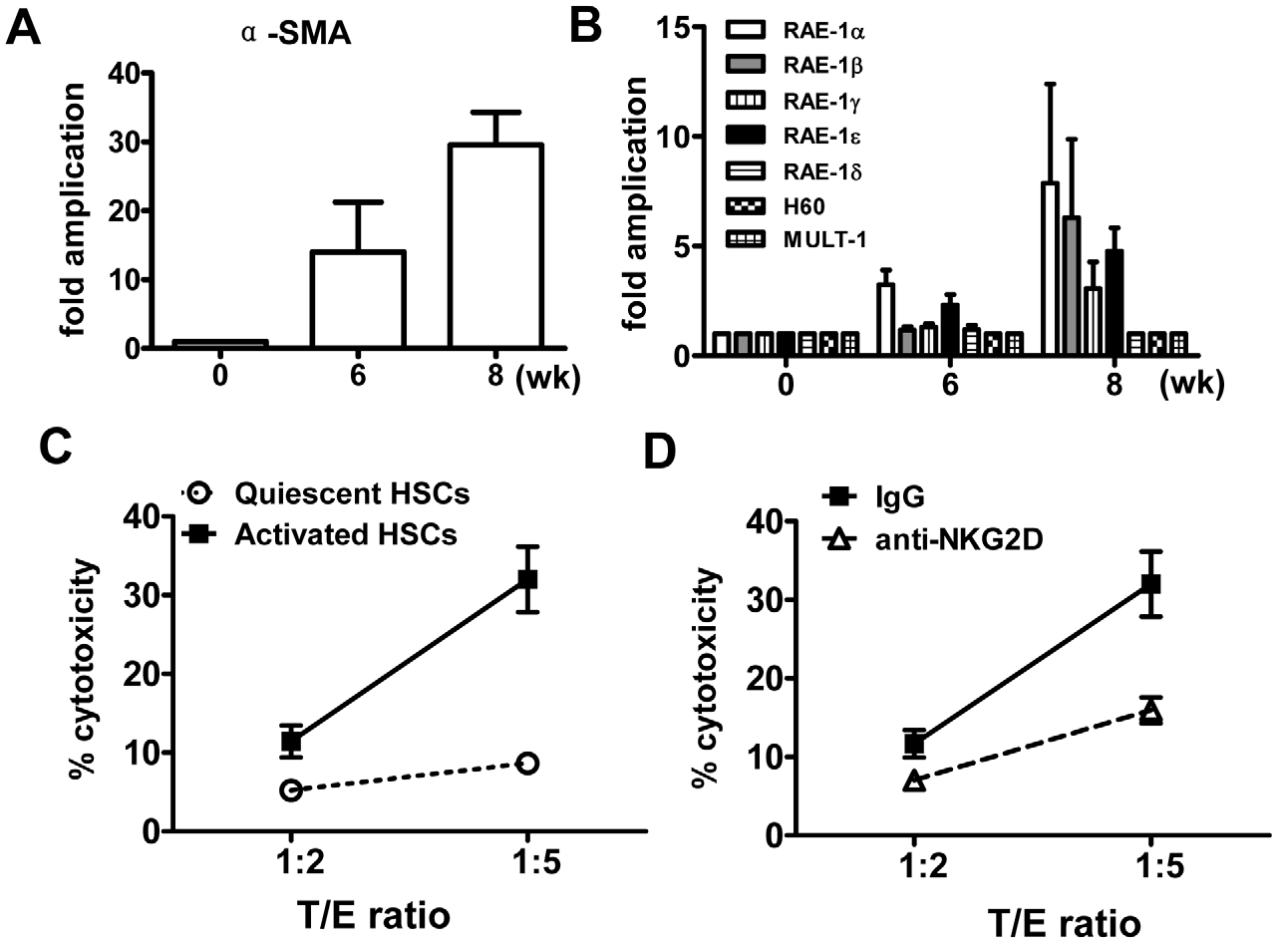
NKG2D/RAE1 interaction is involved in the cytotoxicity of NK cells against activated HSCs. (A, B) HSCs were isolated from *S. japonicum*-infected mice or uninfected mice. Expression of α-SMA (A) and various NKG2D ligands (B) on HSCs was detected by quantitative PCR. Values were normalized to β-actin in the same samples and expressed as fold change in comparison with the samples from uninfected controls (0 wk). Data were shown as mean ± SEM from three independent experiments. (C, D) NK cells isolated from *S. japonicum*-infected mice were used as effector cells. (C) HSCs isolated from normal mice (quiescent HSCs) or 8-week *S. japonicum*-infected mice (activated HSCs) were used as target cells. (D) HSCs isolated from 8-week *S. japonicum*-infected mice (activated HSCs) were used as target cells. A dose of 10 µg/mL anti-NKG2D (blocking) mAb was added in the coculture for the blockade. T/E, target-effector ratio.

Because activated HSCs were more susceptible to hepatic NK cell killing via RAE1/NKG2D recognition [19], [22], we attempted to determine whether NKG2D-ligand interactions were also involved in the suppression of *S. japonicum* egg-induced fibrosis by NK cells. First, the identity of activated HSCs isolated from *S. japonicum*-infected mice was confirmed by the expression of α-SMA (Figure 6A). Next, the expression of NK receptor activating ligands including RAE1, histocompatibility 60 (H60), and UL16-bingding protein like transcript 1(Mult-1) on HSCs was examined. As shown in Figure 6B, the expression of RAE1α, β, γ, and ε was dramatically increased on these activated HSCs at week 6 and week 8 post-infection, while the expression of RAE1δ, H60, and Mult-1 was not induced. To further determine whether NK cells in *S. japonicum*-infected mice kill activated HSCs, *in vitro* cell-mediated cytotoxicity experiments were performed. As shown in Figure 6C, liver NK cells produced about 30% cytotoxicity against activated HSCs from *S. japonicum*-infected mice, but less than 10% cytotoxicity against quiescent HSCs from normal mice. Furthermore, NKG2D blocking mAb diminished the cytotoxicity of NK cells against activated HSCs (Figure 6D). These data suggested that NK cells might inhibit *S. japonicum* egg-induced liver fibrosis via killing activated HSCs.

## Discussion

Infection with the helminth parasite schistosoma accounts for a significant portion of liver fibrosis cases reported each year in humans. Both T cells and B cells have been implicated in regulating *S. mansoni* egg-induced liver fibrosis. *S. mansoni* egg deposition leads to the development of Th2 response, which promotes the development of liver fibrosis [4], [23], [24]. B cells promote Th2-type response to the *S. mansoni* eggs, thus B-cell-deficient mice display an increased hepatic fibrosis at 10 weeks post-infection [25], [26]. NK cells are an important population of innate immune cells in the liver, comprising 30%–40% and 10%–20% of total intrahepatic lymphocytes in humans and mice, respectively [27], [28]. However, the roles of NK cells in schistosoma egg-induced liver fibrosis remain obscure. Treatment with anti-NK1.1 Abs to deplete NK1.1+ cells, which include NK cells (CD3-NK1.1+) and NKT (CD3+NK1.1+) cells, enhanced *S. mansoni*-induced liver fibrosis, thus suggesting that NK cells may be involved in negative regulation of schistosoma egg-induced liver fibrosis [29].

In this study, we provided several lines of evidence suggesting that NK cells negatively regulated *S. japonicum*-induced liver fibrosis. First, the absolute number of hepatic NK cells dramatically increased in the liver after *S. japonicum* infection. And these NK cells were activated as demonstrated by the upregulation of activation marker CD69 and production of IFN-γ (Figure 1). Second, depletion of NK cells using anti-ASGM1 Ab accelerated liver fibrosis (Figure 2). Third, injection of poly I∶C enhanced the accumulation of NK cells in the liver and activated NK cells to secrete IFN-γ (Figure 4), attenuating *S. japonicum* egg-induced liver fibrosis (Figure 3). The change in liver fibrosis was not due to differences in the intensity of infection or egg burden because worm pairs, total worms, eggs entrapped in the livers, and granuloma size of all groups in an experiment were identical (data not shown).

Currently, the underlying mechanisms by which NK cells are activated after *S. japonicum* infection are not clear. Our results showed that NK cells were activated before *S. japonicum* worms lay eggs (about 24 days post-infection), because the expression of CD69 on NK cells had been upregulated 3 weeks post-infection. The upregulation of CD69 was enhanced after worms lay eggs (Figure 1). These data suggest that the antigens or secretion of both worms and eggs may be involved in the activation of NK cells. Although NK cells express many activating receptors and toll-like receptors [16], [30], which lead to direct NK cell activation when encountering their ligands. Activation of NK cells by most pathogens seems to be indirect and results from signals provided by accessory cells, such as monocytes, macrophages and dendritic cells [31], [32]. Further studies are needed to determine whether NK cell activation during *S. japonicum* infection requires the presence of accessory cells and how these cells interact.

To investigate the underlying mechanisms by which NK1.1+ cells inhibited *S. mansoni*-induced liver fibrosis, Asseman et al. examined hepatic mRNA expression of IFN-γ as well as serum IFN-γ levels. They did not observe variation between control Ab-treated mice and anti-NKl.l Ab-treated mice. So they did not perform other experiments to study the role of IFN-γ in *S. mansoni*-induced liver fibrosis [29]. We also examined serum level of IFN-γ in *S. japonicum*-infected mice, but the level was too low to detect. Then we examined IFN-γ production from their hepatic mononuclear cell cultures, and found that IFN-γ secretion by hepatic MNCs from anti-ASGM1 Ab pretreated mice was strongly reduced compared to that from control IgG pretreated mice (Figure 1E). These data suggested that NK cells were the primary source of IFN-γ after *S. japonicum* infection. Furthermore, disruption of the IFN-γ gene accelerated *S. japonicum* egg-induced liver fibrosis and reduced poly I∶C-mediated suppression of liver fibrosis (Figure 5). Therefore, it is postulated that the production of IFN-γ by NK cells after *S. japonicum* infection was an important mechanism responsible for NK cell-mediated suppression of liver fibrosis. It is reported that IFN-γ also has high anti-fibrogenic activities and immune protection in human schistosomiasis [33], [34].

It is well known that IFN-γ promotes Th1 cell differentiation while dampening Th2 cell expansion [7], [8]. Thus there might be more Th2 cells in IFN-γ−/− mice. However, despite the absence of endogenous IFN-γ, Th2 cytokine (IL-4, IL-5, and IL-13) production in response to soluble egg antigen (SEA) stimulation by splenocytes was not significantly altered in IFN-γ−/− mice compared to wild mice [35], [36]. And we also found that IL-4, IL-5, and IL-13 mRNA levels in the liver of IFN-γ−/− mice were similar to those of wild mice (Figure 5C). So the increased fibrosis in IFN-γ−/− mice may be not because that IFN-γ−/− mice had higher levels of Th2 cytokines. According to other reports, IFN-γ suppression of liver fibrosis was mainly mediated through inducing HSC apoptosis and cell cycle arrest [37].

Although hepatic invariant NKT (iNKT) cells could also produce IFN-γ after transferring live *S. mansoni* eggs into the caecal vein of mice, the contribution of iNKT cells in IL-4 production by liver monocytes was total, whereas that in IFN-γ production was partial and minor [38]. Our data also demonstrated that the contribution of NKT cells in IFN-γ production was less than NK cells (Figure 1). In addition, hepatic NKT cells played a minor role in DDC-induced liver fibrosis, and inhibited CCl_4_-induced liver fibrosis in the early stage but not in the late stage of fibrosis [19], [39]. Thus, we speculated that the suppression of schistosoma egg-driven liver fibrosis by NK1.1 positive cells was mainly dependent on NK cells.

The results presented here also demonstrated that NKG2D-Rae1 interaction might be involved in NK cell-mediated HSC death, which contributed to NK cell suppression of *S. japonicum* egg-induced liver fibrosis. The balance in the expression of activating and inhibitory ligands will determine whether a cell becomes a target for NK cell-mediated killing [40], [41]. RAE1 was previously identified as an NK cell activating ligand to stimulate NK cytotoxicity [42]. Here we showed that the expression of RAE1α, β, γ, and ε was detected at much higher levels on activated HSCs from *S. japonicum*-infected mice compared with that on quiescent HSCs from uninfected mice (Figure 6A, B). Moreover, the expression of several NK receptor inhibitory ligands, including H2-D1, H2-D4, and Clec2D was unchanged on activated HSCs (data not shown). The interaction between RAE1 and NKG2D has been reported to be an important mechanism contributing to the cytotoxicity of NK cells against activated HSCs [19], [22]. Our data also demonstrated that liver NK cells could kill activated HSCs via NKG2D/RAE1-dependent mechanism (Figure 6C, D).

In addition, we examined the role of NK cells in IL-13 expression. Our results showed that depletion of NK cells did not affect mRNA expression of IL-13 in the liver (Data not shown). And the levels of IL-13 in the culture supernatants of hepatic MNCs from anti-ASGM1-treated mice and IgG-treated mice were similar (Data not shown).

In summary, our findings suggested that after *S. japonicum* infection, murine hepatic NK cells were activated and suppressed liver fibrosis mainly via production of IFN-γ and via killing activated HSCs. Because of the difficulty of acquiring liver samples from humans, the function of hepatic NK cells in patients with schistosomiasis is still unknown, and clinical researches only focus on the peripheral blood mononulcear cells (PBMCs). E. Speziali et al. found that NK cells were the major source of IFN-γ when *in vitro* stimulating PBMCs with *S. mansoni* antigens. However, in individuals over 70 years old, infection positive individuals had lower percentage of IFN-γ+ NK cells than negative controls [43]. Other clinical studies showed that the cytotoxicity activity of NK cells was reduced in children infected with *Schistosoma haematobium* and *S. mansoni* compared with healthy controls [44], [45]. Using a mouse model, our study first showed the suppression role of hepatic NK cells in *S. japonicum* egg-induced liver fibrosis. Therefore, stimulation of NK cell activity in schistosoma-infected patients especially in the elderly and children may have beneficial effect in suppressing liver fibrosis.
